# Systems biology for identifying liver toxicity pathways

**DOI:** 10.1186/1753-6561-3-s2-s2

**Published:** 2009-03-10

**Authors:** Zheng Li, Christina Chan

**Affiliations:** 1 Cellular and Molecular Biology Lab, Department of Chemical Engineering and Materials Science, Michigan State University, East Lansing, MI 48824, USA; 2 Department of Biochemistry and Molecular Biology, Michigan State University, East Lansing, MI 48824, USA; 3 Department of Computer Science and Engineering, Michigan State University, East Lansing, MI 48824, USA; 4 Biomedical engineering department, Boston University, Boston, MA, 02215, USA

## Abstract

Drug-induced liver toxicity is one of the leading causes of acute liver failure in the United States, exceeding all other causes combined. The objective of this paper is to describe systems biology methods for identifying pathways involved in liver toxicity induced by free fatty acids (FFA) and tumor necrosis factor (TNF)-α in human hepatoblastoma cells (HepG2/C3A). Systems biology approaches were developed to integrate multi-level data, i.e., gene expression, metabolite profile, toxicity measurements and *a priori *knowledge to identify gene targets for modulating liver toxicity. Targets that modulate liver toxicity, *in vitro*, were computationally predicted and some targets were experimentally validated.

## Background

The liver plays a central role in clearing toxic chemicals from the human body and is susceptible to toxicity during the process. More than 900 drugs have been found to induce liver toxicity, which is a leading cause of acute liver failure in the United States, exceeding all other causes combined [1]. It is one of the most common reasons for drug recalls, resulting in substantial financial cost to the pharmaceutical industry. Different mechanisms are involved in liver toxicity, for example, the disruption of the cellular membrane, alteration of mitochondrial function or drug metabolism pathways, non-specific covalent binding of the drug to the cell's proteins or activation of apoptotic signaling pathways, to name some [1]. Liver toxicity can also be induced by nutrients, e.g. a high fat diet. Elevated Free fatty acid (FFA) levels increase the accumulation of triglycerides in liver cells and enhance the risk of developing non-alcoholic steatohepatitis (NASH), which is characterized by extensive cell death and inflammation [2]. Identifying the pathways that contribute to the development of liver toxicity by drugs or diet may provide insight into minimizing or preventing the toxicity.

We investigated fatty acid induced liver toxicity *in vitro *using HepG2/C3A cells as the experimental model system. Saturated fatty acid, palmitate, was found to induce significantly higher toxicity as compared to unsaturated fatty acids [3]. To elucidate the underlying toxicity pathways, dynamic, multiple-level information, i.e., microarray gene expression, metabolite profile, toxicity measurements and pathways information, were collected. Systems biology approaches were developed thereafter to integrate the aforementioned multi-level data to identify the toxicity pathways. First, dynamic module mapping analysis was applied to study the dynamic changes in the pathways induced by fatty acid treatment. Based upon the dynamic pathways analysis, we hypothesized and confirmed that toxic signals were induced within the first 24 hours (day one). Second, toxicity measurements and gene expression profile on day one were integrated using a Three-Stage-Integrative-Pathway-Search (TIPS^©^) framework. Briefly, toxicity-relevant genes were identified using genetic algorithm coupled partial least squares analysis (GA/PLS) and toxicity pathways were subsequently reconstructed based upon the expression of the identified genes using Bayesian network analysis. The predicted toxicity pathways were then used to infer the effects of perturbing a gene on the liver toxicity using Bayesian inference. Finally, a hierarchical approach was developed to identify toxicity relevant genes by integrating toxicity measurement, metabolite profile, gene expression and pathway information. Gene targets, such as NADH dehydrogenase, were identified and experimentally confirmed to have significant effects on reducing the toxic signal, reactive oxygen species (ROS), and ultimately toxicity levels in palmitate treated liver cells. The details of the approaches discussed in this paper are published elsewhere [4-6]. The objective of this paper is to provide an overall picture of how systems biology approaches may be used to integrate multiple-source information for novel biological discoveries.

## Methods

### Cell culture

One million HepG2/C3A cells were seeded into each well of a 6-well culture plate. Cells were incubated at 37°C and in 10% CO_2 _atmosphere. After the cells reached confluence, the medium was replaced with 2 ml of the chosen medium, either HepG2; or the FFA medium containing 0.7 mM palmitate, oleate or linoleate; or the FFA-TNF-α medium. The FFAs were dissolved in 4% fatty acid-free BSA. TNF-α was added from a 100 μg/ml stock in deionized water to make the desired final concentrations of either 20 or 100 ng/ml.

### Toxicity, gene expression and metabolite measurement

The cytotoxicity of the treatments was measured as the fraction of lactate dehydrogenase (LDH) released into the medium. Cytotoxicity detection kit (Roche Applied Science, Indianapolis, IN) was used to measure the LDH release. The gene expression profiles were obtained with the cDNA microarrays at the Van Andel Institute, Grand Rapids, MI (protocols available online at [7]. The net uptake or production of a metabolite was calculated by the difference in the concentration of the metabolite in the medium, before and after the treatment. The concentrations of metabolites were measured using enzymatic assays or HPLC. The experimental details were described in reference [2].

### Gene module map analysis

Module map analysis [8] was applied to identify the important pathways perturbed by FFA treatment using Genomica (available at [9]). 350 biologically meaningful gene sets were first defined based upon their functional category or pathways defined in the MsigDB database [10]. The number of genes within a gene set that significantly changed under a treatment was obtained and the significance calculated with hypergeometric test as compared to random selection. The module maps at different time points were compared to identify the dynamics of the modules that are important to the cytotoxic phenotype.

### Three-Stage-Integrative-Pathway-Search (TIPS^©^) framework

TIPS^© ^approach was developed to integrate gene expression and toxicity measurement to identify toxicity relevant gene targets and pathways. Three methods, including genetic algorithm coupled partial least squares analysis (GA/PLS), constrained independent component analysis (CICA) and Bayesian network analysis (BN) were integrated within the framework. As a first approximation, we assume a log linear relationship between gene expression and toxicity. In order to extract an independent pathway related to a phenotype, such as cytotoxicity, from the gene expression profile, we applied a constrained ICA (CICA) approach. The relevance of the genes to the toxicity identified by GA/PLS along with the cytotoxicity profiles were used as constraints in CICA. CICA extracted a phenotype-relevant-component from the gene expression data. This was identified by minimizing the mutual information between the phenotype-relevant-component and the other independent components while maximizing the correlation between the component and the constraints. The expression profiles of the genes with the highest weights in CICA were used in BN analysis for network reconstruction. The reconstructed network was perturbed to identify i) which genes, when perturbed, had an impact on altering the cytotoxic phenotype in the palmitate cultures, and ii) how perturbing a gene (node) affected the other genes in the network. More details can be found in our published paper [6]. TIPS^© ^was further extended to identify genes relevant to multiple cellular responses, e.g., multiple metabolites, in a separate study [11].

### Hierarchical approach to integrate multi-level data

A hierarchical framework was developed to integrate the toxicity measurement, metabolic profile and gene expression and pathway information to identify the genes and biological processes that may be involved in the phenotypic responses. The framework consisted of three stages. First, the metabolite changes associated with the cytotoxic phenotype were identified with Fisher's Discriminant Analysis (FDA). To identify the signaling and gene pathways involved in the toxicity, the genomic responses obtained using cDNA microarrays were analyzed with gene set enrichment analysis (GSEA) [10]. Finally, the gene expression and metabolite profiles were integrated with multi-block partial least squares (MBPLS) regression analysis to identify the genes, which were most relevant to the metabolic changes that correlated highly with cytotoxicity. Further details on the hierarchical approach are described in [5].

## Results

### The essential first 24 hours to toxicity

**Figure 1:**
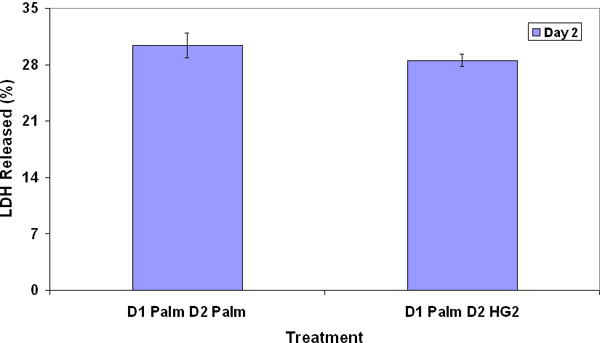
**Effects of palmitate on toxicity in day 2 culture**. The cells were treated first with palmitate for 24 hours and exposed to either control medium (D1 Palm D2 HG2) or palmitate (D1 Palm D2 Palm) in the next 24 hours. Cytotoxicity of the treatments was measured. No significant difference was detected between the two treatments.

Dynamic gene module map analysis results are summarized in Table 1. Palmitate was found to affect only 5 functional pathways on day 1, while it affected significantly more pathways on day 2, which coincided with the higher toxicity observed on day 2 with palmitate treatment. As discussed in reference [4], palmitate down-regulated metabolic pathways such as electron transport chain (ETC) and TCA cycle on day 1. On day 2, palmitate upregulated many cell death related pathways, such as apoptosis, TNF signaling pathway, and down-regulated protective pathways, such as pentose phosphate pathway (PPP) and glutathione pathway. Palmitate did not have significant effects on day 3. Based upon the pathway analysis, we hypothesized that palmitate altered metabolic pathways, such as PPP and TCA, to permit the production of toxic signals on day 1. The toxic signals subsequently caused up-regulation of cell death pathways and down-regulation of protective pathways. To test this hypothesis, we compared the toxicity measurement of cells treated with palmitate for one day with cells treated with palmitate for two days (Figure 1). There was no significant difference between these two treatments, which suggested that palmitate sufficiently induced the toxic phenotype within the first 24 hours of treatment.

**Table 1:** Number of pathways affected by FFA at different time points.

	**Day 1**	**Day 2**	**Day 3**
**Palmitate**	6	39	0
**Oleate**	52	4	0
**Linoleate**	29	9	0

Note that PPP and glutathione pathways were down-regulated on day 2. PPP and glutathione are known to be related to cellular reactive oxygen species (ROS) level. PPP produces NADPH which is required in converting oxidized glutathione to reduced glutathione. Reduced glutathione is used to reduce ROS levels. Therefore we hypothesized that ROS maybe a toxic signal. We confirmed this hypothesis in a separate study [3] by treating the cells with palmitate along with ROS scavengers and found the toxicity indeed reduced significantly.

### Toxicity-relevant network

**Figure 2:**
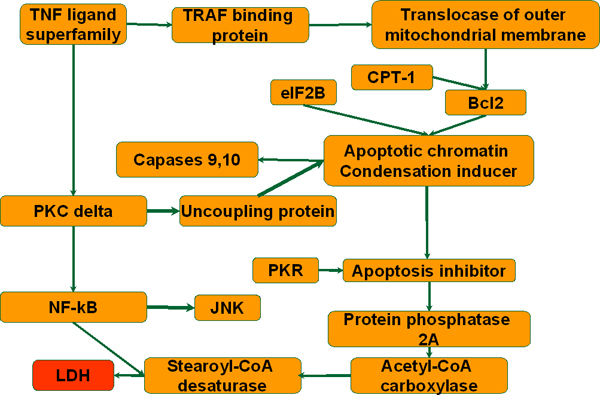
**Simplified toxicity network**. LDH (in red) is the phenotype node and all other nodes are genes predicted to be relevant to toxicity using Bayesian network analysis.

To identify the gene targets that may be perturbed to reduce liver toxicity, we integrated the toxicity measurements with gene expression profile using the TIPS^© ^approach. A simplified toxicity relevant network was reconstructed as shown in Figure 2. A more detailed network is shown in reference [6]. The network was used to predict the effect of perturbing a gene on the liver toxicity. For example, we predicted the probability of a high level of toxicity upon palmiate treatment should be reduced significantly by up-regulating stearoyl-CoA desaturase (SCD). The prediction was experimentally confirmed with SCD activation using two chemical agents, clofibrate and ciprofibrate. Other predictions made by the model are discussed further in reference [6]. In addition, we applied a modified GA/PLS to identify genes relevant to multiple cellular responses, including liver toxicity and Triglyceride (TG) accumulation [11]. The analyses identified NADH dehydrogenase and mitogen activated protein kinases (MAPKs) were relevant to both cytotoxicity and lipid accumulation. Indeed, inhibiting NADH dehydrogenase and c-Jun N-terminal kinase (JNK) reduced cytotoxicity significantly and increased intracellular TG accumulation. In fact much greater reduction in the toxicity was observed upon inhibiting the NADH dehydrogenase or MAPK than for the stearoyl-CoA desaturase (SCD) activation [11], thus suggesting the incorporation of more information, i.e. more metabolites, is beneficial.

### Toxicity relevant gene targets

Motivated to identify more relevant toxicity-related genes using multiple-source information, we developed a hierarchical approach to integrate multi-level data, i.e., toxicity measurements, metabolite profile, gene expression profile with pathway information to identify potential target genes. First we identified toxicity relevant metabolites using discriminant analysis. As a result, ketone bodies, such as acetoacetate and beta-hydroxybutarate, were found to be highly relevant to the toxic phenotype. Second, we identified toxicity relevant gene sets with GSEA analysis. We found gene sets, such as ROS, ETC, PPP and fatty acid metabolism were significantly enriched. Finally, MBPLS was applied to identify individual genes that were relevant to the aforementioned metabolites and in turn toxicity. Genes, such as glutathione S-transferase, NADH dehydrogenase and ALDH1A1, were identified to be relevant based upon their regression coefficients. NADH dehydrogenase and ALDH1A1 were experimentally confirmed to have significant effects on the ROS as well as the toxicity levels. Further details of these results can be found elsewhere [5].

## Discussion

It is the objective of this paper to illustrate how biological findings can be derived from one or more data sources. We first demonstrated that integrating dynamic gene expression profile with pathway information helped to identify the dynamic changes in the pathways and derived hypothesis for further experimental testing. After identifying the timing of the events, we integrated gene expression profile with toxicity measurements using the TIPS^© ^approach to first identify toxicity relevant genes and then reconstructed a network based upon the expression levels of those genes. The TIPS^© ^approach provided a way to reconstruct context specific pathways using a limited number of microarray data. It provided an alternative method for pathway to network reconstruction based upon interaction measurements and genome wide network perturbations. It also provided a predictive framework to construct hypotheses based upon computational inference of virtual perturbations. However we would also like to point out that this study is based upon data from an in vitro system, namely HepG2 cells. Thus the insights gained from the analysis could be quite different from what takes place in vivo, i.e., in the liver. In vivo study would be necessary to derive biological insights of this kind.

We also illustrated that integrating more information improved the ability of the computational model to identify relevant gene targets and predict possible effects upon perturbation. Within the hierarchical framework, incorporating information, such as metabolite profiles and pathway information, identified genes and pathways that were induced by a toxic signal, such as ROS. Perturbing the genes identified by the multi-source data provided more relevant targets of toxicity as compared with the genes identified with single source, i.e. gene expression, and toxicity measurements. Integrating other sources of information, such as sequence information, could further improve the modeling capabilities. For example, sequence information, such as single nucleotide polymorphism (SNP), has recently been successfully integrated with gene expression data using eQTL and Bayesian network analysis to identify disease related genes [12].

## Conclusion

In conclusion, it is feasible to identify phenotype relevant genes using data driven systems biology approaches. Incorporating more information in an effective manner, i.e., hierarchical approach, could improve both target identification and phenotype prediction.

## List of abbreviations used

BN: Bayesian network analysis; CICA constrained independent component analysis; ETC: electron transport chain; FFA: free fatty acid; GA/PLS: genetic algorithm coupled partial least squares analysis; GSEA: gene set enrichment analysis; MBPLS: multi-block partial least squares; NASH: non-alcoholic steatohepatitis; PPP: pentose phosphate pathway; ROS: reactive oxygen species; SCD: stearoyl-CoA desaturase; SNP: single nucleotide polymorphism; TG: triglyceride; TIPS: Three-Stage-Integrative-Pathway-Search; TNF: tumor necrosis factor.

## Competing interests

The authors declare that they have no competing interests.

## Authors' contributions

ZL conceived the methodology and performed part of the experiments and wrote the manuscript. CC conceived the study and supervised the experiment and writing of the manuscript. All authors read and approved the final manuscript.
